# Formulation and *In Vitro* Evaluation of Ofloxacin Tablets using Natural Gums as Binders

**DOI:** 10.3797/scipharm.1401-14

**Published:** 2014-03-10

**Authors:** Amisha K. Mistry, Chirag D. Nagda, Dhruti C. Nagda, Bharat C. Dixit, Ritu B. Dixit

**Affiliations:** 1 Ashok & Rita Patel Institute of Integrated Study & Research in Biotechnology and Allied Sciences, New Vallabh Vidyanagar-388120, Affiliated to Sardar Patel University, Vallabh Vidyanagar-388120, India.; 2 Indukaka Ipcowala College of Pharmacy, New Vallabh Vidyanagar-388315, India.; 3 A. R. College of Pharmacy & G. H. Patel Institute of Pharmacy, Vallabh Vidyanagar-388120, India.; 4 Chemistry Department, V. P. & R. P. T. P. Science College, Vallabh Vidyanagar-388120, India.

**Keywords:** Tablet, Natural binders, Physico-chemical parameters, Release kinetics, Similarity factor

## Abstract

Natural gums are economical, easily available, and useful as tablet binders. In the present investigation, an attempt was made to formulate Ofloxacin tablets using three natural binders, namely Acacia arabica, Hibiscus esculentus, and xanthan gum. Such six batches of Ofloxacin tablets were prepared by using different types and amounts of the natural binders by the wet granulation method. The tablets were analyzed for their hardness, friability, and weight variation, and *in vitro* release was performed in a phosphate buffer at pH 6.8. The prepared tablets were also evaluated for their various release kinetics and similarity factors f_2_. The physical properties of the tablets containing the natural binders showed sufficient hardness, desirable disintegration time, and low friability. Their better percentage of drug release was observed as compared to the marketed formulation showing more than 85% drug release within 45 minutes. The *in vitro* release data was well-fitted into zero-order and the values of release exponent ‘n’ were between 0.303 and 0.514. The high similarity factor f_2_ of 64.50 was achieved with the best batch in comparison to the marketed tablets. The results obtained indicated that the gum Acacia arabica performed as well as gelatin compared to the other binders for the Ofloxacin tablet formulation.

## Introduction

Due to their portability and convenience, tablets are the most widely prescribed dosage forms in the world. One major class of excipients that is used to improve tablet formulations is pharmaceutical binders, sometimes referred to as adhesives [1]. Binders are pharmaceutical excipients that are commonly employed in tablet formulations to impart cohesion on the powder mixture and hence, improve the flow properties on the granules. Binders act by causing aggregation of powders, thereby forming granules through the process of granulation [2]. Natural polysaccharides are widely used in the pharmaceutical and food industries as excipients and additives due to their low toxicity, biodegradability, bio-competitiveness, availability, and low cost. Natural binders like different starches, gums, mucilages, and dried fruits possess binding capacity as well as some other properties like being a disintegrant, filler, and having sustained release. These natural polymers are much safer and more economical than polymers like polyvinylpyrrolidone (PVP) [3, 4]. *Acacia arabica* (Lam.) Willd (Family: Mimosaceae) is commonly known as babul, kikar, or Indian gum. The Arabic tree has been recognized worldwide as a multipurpose tree and it is widely distributed throughout the arid and semi-arid zones of the world [5]. *Hibiscus esculentus L*. (Family: Malvaceae), commonly known as bhindi in India, krajiab kheaw in Thailand, okra plant, kopi arab, kacang bendi, and bhindi in Southeast Asia, is a tropical to subtropical plant that is widely distributed across Africa to Asia, Southern Europe, and America. Okra plays an important role in the human diet by supplying fat, protein, carbohydrate, minerals, and vitamins. Moreover, its mucilage is suitable for certain medical and industrial applications [6]. Xanthan gum is another natural, biosynthetic, edible gum, and an extracellular polysaccharide produced by the bacterium Xanthomonas campestris. Xanthan gum consists of glucose, mannose, and glucuronic acid, and is used in different foods as a thickener and stabilizer [7].

Ofloxacin is a synthetic chemotherapeutic antibiotic of the fluoroquinolone class considered to be a second-generation fluoroquinolone. It is used to treat mild-to-moderate urinary tract infections, prostatitis, lower respiratory tract infections, and skin infections [8]. The aim of the present work is to prepare tablets using three different types of natural polymers, namely Acacia arabica, Hibiscus esculentus, and xanthan gum, and to evaluate these formulations for different physical parameters. The influence of different types and amounts of binders on the tablet quality (i.e. crushing strength, friability, disintegration time, and dissolution time) were investigated.

## Experimental

### Materials

Ofloxacin was obtained as a gift sample from Loba, Pharmaceuticals and Chemicals Ltd, Mumbai. Carboxymethyl cellulose, dicalcium phosphate, magnesium stearate, talc, and other chemicals were procured from Loba, Pharmaceuticals and Chemicals Ltd, Mumbai.

Acacia arabica; Hibiscus esculentus; Xanthan gum were procured from a local market. OF^®^ (marketed formulation) was purchased from a local market.

### Methods

#### Preparation of Tablets

Ofloxacin tablets were prepared by the wet granulation technique [9]. All the ingredients were grinded properly using a mortar and pestle. The composition of different batches for the preparation of the tablets using different binders with a fixed amount of the drug is shown in Table 1. The required quantity of the drug, binder, disintegrate, and diluents were passed through a # 40 sieve separately and then mixed uniformly by methanol as the granulating agent to get a coherent wet granulate which was screened through # 16 sieve to obtain coarse granules, followed by drying of the granules at 45°C for 1 hour. The dried granules were then passed through the # 20 sieve and were lubricated with magnesium stearate and talc. Finally, the dried granules were compressed as tablets by using the Mini Press compression machine.

#### Characterization of the Tablet

The prepared tablets were evaluated for various parameters like physical appearance, hardness, friability, and disintegration time according to the USP 29 requirements [10, 11] and are shown in Table 2. The tablets were evaluated for hardness by using a Monsanto hardness tester. The hardness reported is an average of three measurements. Twenty tablets were weighed and placed in a friabilator. After rotating for 4 min, that is 100 revolutions, the percentage of weight loss was determined as an indicator of friability. The disintegration test was performed in water at 37°C. The disintegration time reported is an average of three determinations.

#### In Vitro Dissolution Study

**Fig. 1:**
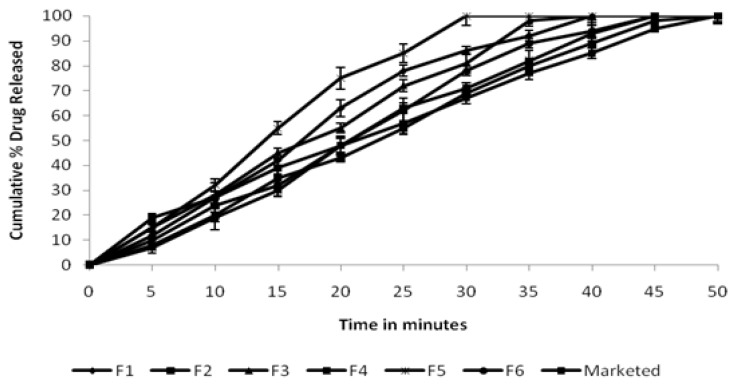
*In vitro* release profile of Ofloxacin from tablets containing different natural binders Values are mean ± SD (*n* = 3)

The dissolution rate of Ofloxacin from the various tablets was studied using the USP XXIII six-station dissolution test apparatus (Electrolab, INDIA) with a paddle stirrer. The dissolution rate was studied by placing one tablet containing 300 mg Ofloxacin in 900 ml of a phosphate buffer of pH 6.8 maintained at 37±0.5°C with a speed of 50 rpm. Samples of 5 ml were withdrawn at different time intervals, filtered (though 0.45 μm), and replaced with 5 ml of fresh dissolution medium. The samples were properly diluted and estimated spectrophotometrically at 207 nm by using the ELICO double beam UV spectrophotometer [12]. The *in vitro* release profile of Ofloxacin tablets containing different natural binders is shown in Figure 1.

#### Data Analysis

The results of the *in vitro* drug release study were fitted with various kinetic equations like zero-order (% release vs time), first-order (log % unreleased vs time), and the Higuchi matrix (% release vs square root of time). In order to define a model which will represent a better fit for the formulation, the drug release data was further analyzed by the Peppas equation, M**_t_**/M_∞_ = k t^n^, where M**_t_** is the amount of drug released at time ‘t’ and M_∞_ is the amount released at time ‘∞’, the M**_t_**/M_∞_ is the fraction of drug released at time ‘t’, ‘k’ is the kinetic constant and ‘n’ is the diffusional exponent, a measure of the primary mechanism of drug release. R^2^ values were calculated for the linear curves obtained by regression analysis of the above plots [13]. The similarities between the two dissolution profiles were assessed by a pair-wise model independent procedure such as the similarity factor (f_2_) [14]:

$${\text{f}}_{2}=50\times \text{log}\left\{{\left[\left(1+\frac{1}{\text{n}}\right)\sum_{\text{t}=1}^{\text{n}}{\left({\text{R}}_{\text{t}}-{\text{T}}_{\text{t}}\right)}^{2}\right]}^{-0.5}\times 100\right\}$$

Where, ‘n’ is the number of pull points, w_t_ is an optional weight factor, R_t_ is the reference profile at time point ‘t’, and T_t_ is the test profile at the same time point; the value of f_2_ should be between 50 and 100. An f_2_ value of 100 suggests that the test and reference profiles are identical and, as the value becomes smaller, the dissimilarity between release profiles increases [15]. The above values are summarized in Table 3.

## Results and Discussion

A large number of natural polymers have been used in pharmaceutical preparations. Natural substances like starches, mucilages, gums, and dried fruits have been used as binding agents. In the present study, three natural binders, namely Acacia arabica, Hibiscus esculentus, and xanthan gum, were used to prepare Ofloxacin tablets. Tablets were prepared with two different amounts of binders and evaluated for different physicochemical parameters as shown in Table 2. It can be seen from the results that as the amount of binder increased, the hardness and disintegration times increased, and the friability values decreased in the tablets. This finding may be attributed to the gel-forming property of the gum present in the tablet matrix in line with the similar results reported earlier [1, 16]. The hardness of the tablet varied between 22 and 45 N, clearly indicating that they were strong tablets and could withstand the mechanical shocks. This is combined with the friability (less than 1%) of all the formulations, which demonstrated the effecttiveness of the gum for its use as a binder. In general, the tablet hardness was between 40–50 Newton, the friability less than 1%, and the disintegration time less than 30 minutes, which was within pharmacopoeial limits [17]. It was observed from Table 2 that all formulations were within acceptable levels [10].

The dissolution profile of the prepared tablets is shown in Figure 1. The drug release profiles were found to be similar, despite the varying type of binder and physicochemical characteristics of the excipients. It was found that the drug release was decreased with an increase in the concentration of the gum. All the batches indicated a better drug release profile and showed more than 85% within 45 min from the tablets prepared using different types and amounts of the binders.

The release exponent ‘*n’* and R_2_ values for the formulations are given in Table 3. It can be seen from the data analysis of the release profiles according to the different kinetic models that the highest correlation was observed with zero-order as compared to the other models. The tabulated data showed that values of ‘n’ were between 0.303 and 0.514. This implies that the release mechanism is Fickian and not much variation was observed in the ‘n’ value.

**Fig. 2:**
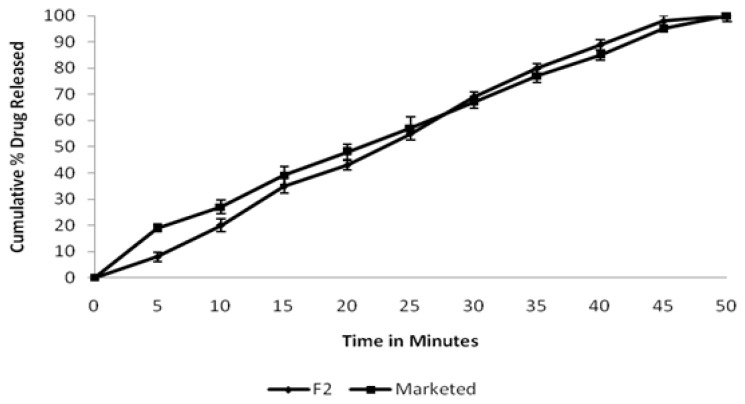
Comparison of *in vitro* release profiles of selected batch (F2) with the marketed tabletValues are mean ± SD (*n* = 3)

Among all of the formulations, F2 showed the best physicochemical parameters and release profile as compared to the other formulations. The release profile of the selected batch was compared with the marketed preparation (OF^®^) as shown in Figure 2. It can be observed that the release profile of the tablets prepared using Acacia arabica as a natural binder have similar release characteristics to that of the commercially available formulation. The similarity factor *f_2_* was a logarithmic transformation of the sum-squared error of differences between the experimental drug release T**_t_** and the ideal release R**_t_** for over all time points ‘n’. The similarity factor fit the result to be between 50 and 100. It approached ‘0’ as the dissimilarity of the test and the reference profile increased, whereas it attained 100 when the test and reference profile were identical [18]. In the present study, the value for the similarity factor (*f*_2_) for all of the prepared batches was observed to be in the range of 33 to 64. It is evident from the results that batches F1, F3, and F5 did not fulfill the above criteria. The highest value was observed with batch F2 (64.50) which was more comparable to that of the marketed formulation, suggesting that the dissolution profile of the selected formulation (F2) and marketed formulation are similar.

## Conclusion

Ofloxacin tablets were successfully prepared using three different natural binders (Acacia arabica, Hibiscus esculentus, and xanthan gum) which were evaluated for their physicochemical parameters and drug release studies. Among the studied natural binders, *Acacia arabica* was more comparable to Hibiscus esculentus and xanthan gum in terms of drug release and similarity factor in relation to the marketed formulation. Hence, it was concluded that *Acacia arabica* could be used as a binding agent for the formulation of the Ofloxacin tablet. Further, Acacia arabica can substitute more expensive binders. Therefore, *Acacia arabica* as a natural material can be widely used in the field of drug delivery, because it is readily available, cost-effective, eco-friendly, capable of a multitude of modifications, potentially degradable, and compatible due to its natural origin.

## Figures and Tables

**Tab. 1:** Composition of Ofloxacin tablets containing different gums as the binder

Ingredients	Formulations (mg)					

	F1	F2	F3	F4	F5	F6
Ofloxacin	200	200	200	200	200	200
Acacia arabica	7.5	15	–	–	–	–
Hibiscus esculentus	–	–	7.5	15	–	–
Xanthan gum	–	–	–	–	7.5	15
Carboxymethyl cellulose	15	15	15	15	15	15
Dicalcium phosphate	74	66.5	74	66.5	74	66.5
Magnesium stearate	1.0	1.0	1.0	1.0	1.0	1.0
Talc	2.5	2.5	2.5	2.5	2.5	2.5

Total weight of each tablet = 300 mg.

**Tab. 2:** Evaluation of Various Parameters of Ofloxacin Tablets

Batch	Thickness (mm)	Diameter (cm)	Weight variation (mg)	Hardness (N)	Friability (%)	Disintegration time (min)
F1	4.054 ± 0.04	0.89 ±0.09	0.3015 ± 0.01	29.421 ± 3.4	0.3330 ± 0.02	21 ± 2.2
F2	4.051 ± 0.02	0.89 ± 0.09	0.2987 ± 0.02	39.23 ± 2.1	0.2212 ± 0.01	16 ± 1.3
F3	4.052 ± 0.07	0.89 ± 0.08	0.3017 ± 0.01	22.77 ± 2.7	0.3333 ± 0.02	13 ± 2.2
F4	4.055 ± 0.03	0.89 ± 0.07	0.3008 ± 0.01	34.16 ± 3.1	0.3101 ± 0.02	21 ± 1.7
F5	4.055 ± 0.04	0.89 ± 0.10	0.2995 ± 0.01	41.12 ± 3.3	0.3332 ± 0.02	25 ± 1.6
F6	4.053 ± 0.02	0.89 ± 0.07	0.3017 ± 0.02	45.23 ± 3.5	0.2315 ± 0.01	32 ± 2.4

All values are expressed as mean ± SD, n = 3.

**Tab. 3:** Various parameters of the model equations and similarity factor on the *in vitro* release kinetics

Kinetic Model	F1	F2	F3	F4	F5	F6	Marketed Tablet
Zero-Order	0.92	0.99	0.941	0.979	0.851	0.97	0.991
Higuchi	0.83255	0.972	0.9531	0.987	0.9012	0.8742	0.9621
First-order	0.973	0.822	0.7661	0.913	0.976	0.9242	0.87
Peppas	0.807	0.877	0.861	0.849	0.74	0.875	0.946
‘n’ value	0.303	0.434	0.596	0.428	0.49	0.514	0.47
f_2_ Value	43.21	64.50	46.10	57.31	33.72	55.62	–
